# The LDL receptor-related protein 1 (LRP1) facilitates ACE2-mediated endocytosis of SARS-CoV2 spike protein-containing pseudovirions

**DOI:** 10.1016/j.jbc.2025.110227

**Published:** 2025-05-09

**Authors:** Mashhood M. Wani, Joanna M. Cooper, Mary Migliorini, Dudley K. Strickland

**Affiliations:** 1 The Center for Vascular and Inflammatory Diseases, University of Maryland School of Medicine, Baltimore, Maryland, USA; 2 Department of Physiology, University of Maryland School of Medicine, Baltimore, Maryland, USA; 3 Department of Surgery, University of Maryland School of Medicine, Baltimore, Maryland, USA

**Keywords:** ACE2, biosensor, cell surface receptor, lipoprotein receptor-related protein (LPR), LRP1, receptor endocytosis, SARS-CoV-2, virus entry

## Abstract

Severe acute respiratory syndrome coronavirus 2 (SARS-CoV-2), the causative agent of COVID-19, employs the viral spike (S) protein to associate with host cells. While angiotensin-converting enzyme 2 (ACE2) is a major receptor for the SARS-CoV-2 spike protein, evidence reveals that other cellular receptors may also contribute to viral entry. We interrogated the role of the low-density lipoprotein receptor-related protein 1 (LRP1) in the involvement of SARS-CoV-2 viral entry. Employing surface plasmon resonance studies, we demonstrated high-affinity binding of the trimeric SARS-CoV-2 spike protein to purified LRP1. Further, we observed high-affinity interaction of the SARS-CoV-2 spike protein with other low-density lipoprotein receptor (LDLR) family members as well, including LRP2 and the very low-density lipoprotein receptor (VLDLR). Binding of the SARS-CoV-2 spike protein to LRP1 was mediated by its receptor-binding domain (RBD). Several LRP1 ligands require surface exposed lysine residues for their interaction with LRP1, and chemical modification of lysine residues on the RBD with sulfo-NHS-acetate ablated binding to LRP1. Using cellular model systems, we demonstrated that cells expressing LRP1, but not those lacking LRP1, rapidly internalized purified ^125^I-labeled S1 subunit of the SARS-CoV-2 spike protein. LRP1-mediated internalization of the ^125^I-labeled S1 subunit was enhanced in cells expressing ACE2. By employing pseudovirion particles containing a murine leukemia virus core and luciferase reporter that express the SARS-CoV-2 spike protein on their surface, we confirmed that LRP1 facilitates ACE2-mediated psuedovirion endocytosis. Together, these data implicate LRP1 and perhaps other LDLR family members as host factors for SARS-CoV-2 infection.

COVID-19 is a global pandemic that is still having severe effects on both individual lives and economies around the world. This disease is characterized by a broad spectrum of clinical syndromes, ranging from asymptomatic or mild influenza-like symptoms to severe pneumonia and acute respiratory distress syndrome leading to death. Additionally, COVID-19 is associated with both acute and long-term symptoms. The causative agent for COVID-19 is infection with SARS-CoV-2, a member of the coronavirus family.

A significant amount of work has resolved several aspects of SARS-CoV-2 infectivity. Initial entry of the virus into the host cells occurs in two critical steps (for review, see (1)). First, the virus associates with the cell surface through recognition of a receptor-binding domain (RBD) located within the S1 subunit of the virus spike protein by cellular receptors. The host receptor for this family of coronaviruses is angiotensin-converting enzyme II receptor, ACE2 (2, 3, 4). Second, two proteolytic events occur that result in conformational changes in the S2 subunit of the spike protein to form the viral fusion machinery. The first proteolytic event occurs within the S1/S2 boundary, which in the case of SARS CoV-2 is mediated by furin (5) and occurs during virus assembly. A second cleavage at the S2′ site within the S2 subunit of the spike protein generates a conformation change that forms the viral fusion machinery and is catalyzed *via* transmembrane serine protease 2 (TMPRSS2) (6) or by cathepsin L following endocytosis of virions into target cell endosomes (7). Other proteases may also be involved in this activation event (8).

Despite significant advances in our understanding of SARS-CoV-2 infectivity, questions remain about additional host factors that facilitate cell entry of viral particles, and to date several host factors/auxiliary receptors have been identified that facilitate SARS-CoV-2 endocytosis (for review, see (9, 10)). Recent studies have provided evidence suggesting that the LDL receptor-related protein 1 (LRP1) may also function as an auxiliary host factor for SARS-CoV-2 (11). LRP1 is an endocytic and signaling receptor that contains a large ectodomain that interacts with numerous ligands, a transmembrane domain and a cytoplasmic domain which contains two NPxY motifs and two dileucine motives which serve as endocytic and signal transduction motifs (for review see (12)). LRP1 has been identified as a host entry factor for Rift Valley fever virus, (11, 13), and is implicated in the infectivity of vesicular stomatitis virus (VSV) (14). Structurally, LRP1 is a member of the LDL receptor family which includes seven core family members: LDL receptor (LDLR), very low-density lipoprotein receptor (VLDLR), LRP8, LRP4, LRP1, LRP1b, LRP2/gp330. Beyond these core family members, there are also some distant members of the family that include: LRP5/6, SORL1, LRP3, LRP12, LRP10 and LDLRAD3, a small receptor that along with the VLDLR mediates cellular entry of the Venezuelan equine encephalitis virus (15, 16).

In the current investigation, we sought to test the hypothesis that LRP1 may function as a co-receptor with ACE2 to facilitate viral entry of SARS-CoV-2. Using surface plasmon resonance technology and cellular-mediated endocytosis experiments, we confirm high-affinity binding of purified trimeric spike protein to LRP1 as well as LRP1-mediated endocytosis of the recombinant spike protein. By employing pseudovirions expressing the SARS-CoV-2 spike protein, we confirm that LRP1 facilitates ACE2-mediated pseudovirion endocytosis. These data support a role for LRP1 in facilitating the endocytosis of SARS-CoV-2 into cells.

## Results

### LRP1 directly binds the SARS-CoV-2 spike protein

**Figure 1:**
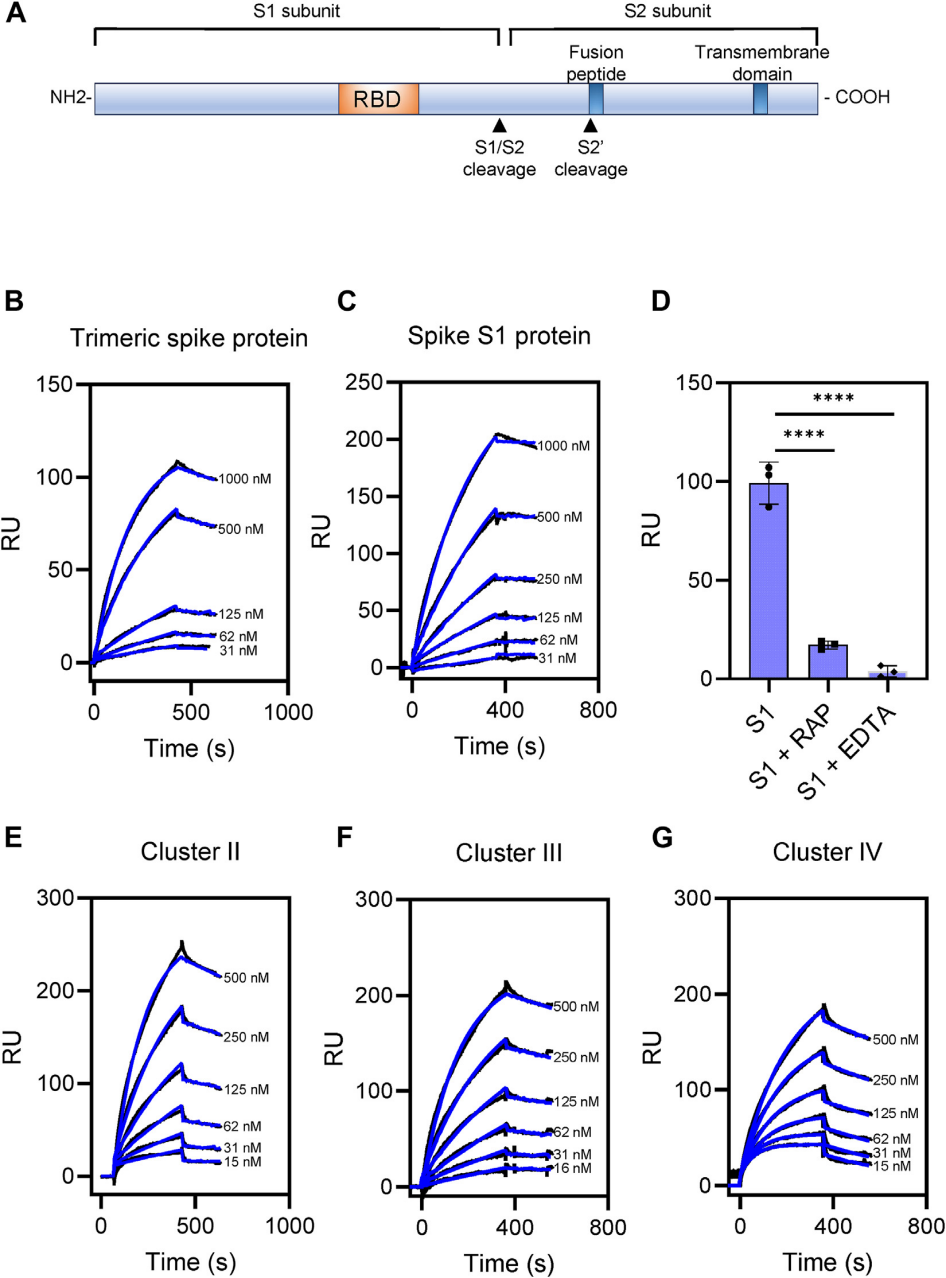
**Trimeric spike protein and S1 subunit from SARS-CoV-2 bind tightly to LRP1.***A*, organization of the SARS-CoV-2 spike protein showing the furin cleavage site (S1/S2) and the S2′ activation cleavage site; RBD, receptor binding domain. *B* and *C*, increasing indicated concentrations of SARS-CoV-2 trimeric spike protein (*B*) or S1 domain of spike protein (*C*) were flowed over LRP1 coupled Biacore CM5 sensor chip. *D*, 500 nM S1 subunit of SARS-CoV-2 spike protein flowed over LRP1-coated CM5 sensor chip in the absence of EDTA or RAP, or in the presence of EDTA or 1 μM RAP. *E*–*G*, increasing indicated concentrations of SARS-CoV-2 S1 subunit flowed over CM5 sensor chips coated with LRP1 cluster II (*E*), cluster III (*F*) or cluster IV (*G*). Fits of the experimental data (*black lines*) to a 1:1 Langmuir binding model are shown as *blue lines*. The data are representative of three independent experiments. *D*, one-way ANOVA with Tukey’s multiple comparison test, ∗∗∗∗*p* < 0.0001.

To test the hypothesis that the SARS-CoV-2 spike protein is recognized by LRP1, surface plasmon resonance experiments were performed. The SARS-CoV-2 spike protein is proteolytically processed to generate an S1 and S2 subunit (Fig. 1*A*) (1). We used surface plasmon resonance (SPR) to characterize the interaction of the SARS-CoV2 spike protein with LRP1. All SPR experiments in these studies were performed in triplicate. Initially, we examined the binding of the trimeric spike protein to LRP1-coated chips, using a trimeric spike protein that was engineered to prevent proteolytic activation (17). The results of this experiment show increased binding of the purified trimeric spike protein to LRP1 as its concentration is increased (Fig. 1*B*). The binding was fit to a 1:1 Langmuir model and is characterized by a relatively slow association rate (4.8 ± 0.3 × 10^3^ M^−1^ s^−1^) and a slow dissociation rate (3.4 ± 0.4 X 10^−^^4^ s^−1^) with a K_D_ value of 72 ± 14 nM (Table 1). We next quantified the binding of purified S1 subunit of the spike protein to immobilized LRP1. The results (Fig. 1*C*, Table 1) confirm high-affinity binding of this region of the SARS-CoV-2 spike protein to LRP1 with association and dissociation rates close to those observed for the trimeric spike protein generating a K_D_ value of 38 ± 18 nM. To confirm the specificity of the interaction of the SARS-CoV-2 S1 subunit to LRP1, we performed a competition experiment in the presence of receptor-associated protein (RAP), an endogenous chaperone protein found in the endoplasmic reticulum that binds tightly to LRP1 and competitively prevents ligands from associating with this receptor (18, 19). The results confirm that the association of RAP with LRP1 prevented SARS-CoV-2 S1 subunit from binding to LRP1 (Fig. 1*D*), revealing the specificity of the interaction. As a second confirmation of binding specificity, we included EDTA in the buffer to remove structural calcium from LRP1 ligand binding repeats. The results reveal that this treatment abolished binding of the SARS-CoV-2 spike S1 subunit to LRP1 (Fig. 1*D*).

**Table 1:** Kinetic constants for the binding of the SARS-CoV-2 spike protein to LDL receptor family members

Ligand	Receptor	k_a_ (M^−1^s^−1^)^a^	k_d_ (s^−1^)^b^	K_D_ nM
Trimeric spike protein	LRP1	4.8 ± 0.3 × 10^3^	3.4 ± 0.4 × 10^−4^	72 ± 14
Spike S1 subunit	LRP1	5.4 ± 1.1 × 10^3^	2.2 ± 1.2 × 10^−4^	38 ± 18
Spike S1 subunit	LRP1 Cluster II	1.3 ± 0.1 × 10^4^	4.2 ± 0.9 × 10^−4^	33 ± 6
Spike S1 subunit	LRP1 Cluster III	1.2 ± 0.1 × 10^4^	2.7 ± 1.3 × 10^−4^	22 ± 12
Spike S1 subunit	LRP1 Cluster IV	9.8 ± 1.0 × 10^3^	6.6 ± 1.1 × 10^−4^	67 ± 10
Spike S1 subunit	LRP2	9.4 ± 1.6 × 10^3^	3.6 ± 0.3 × 10^−4^	40 ± 10
Spike S1 subunit	VLDLR	1.1 ± 0.5 × 10^5^	2.7 ± 0.4 × 10^−4^	5 ± 1
Soluble ACE2	LRP1	5.8 ± 0.6 × 10^3^	2.6 ± 0.4 × 10^−4^	44 ± 6

a Data were analyzed by fits to a 1:1 Langmuir model.

b All data are shown as mean ± STD; n = 3 independent experiments in all cases.

The LRP1 ligand-binding regions are mostly localized to clusters of ligand-binding repeats, termed clusters I-IV, with most ligands binding to clusters II, III, or IV. We examined the binding of the SARS-CoV-2 S1 subunit to clusters II, III, and IV. The results of these experiments revealed that the SARS-CoV-2 S1 subunit binds all clusters, with K_D_ values of 33 ± 6 nM, 22 ± 12 nM, or 67 ± 10 nM for Clusters II, III, or IV, respectively (Fig. 1, *E*–*G*, Table 1).

### SARS-CoV-2 spike protein binds other members of the LDL receptor family

**Figure 2:**
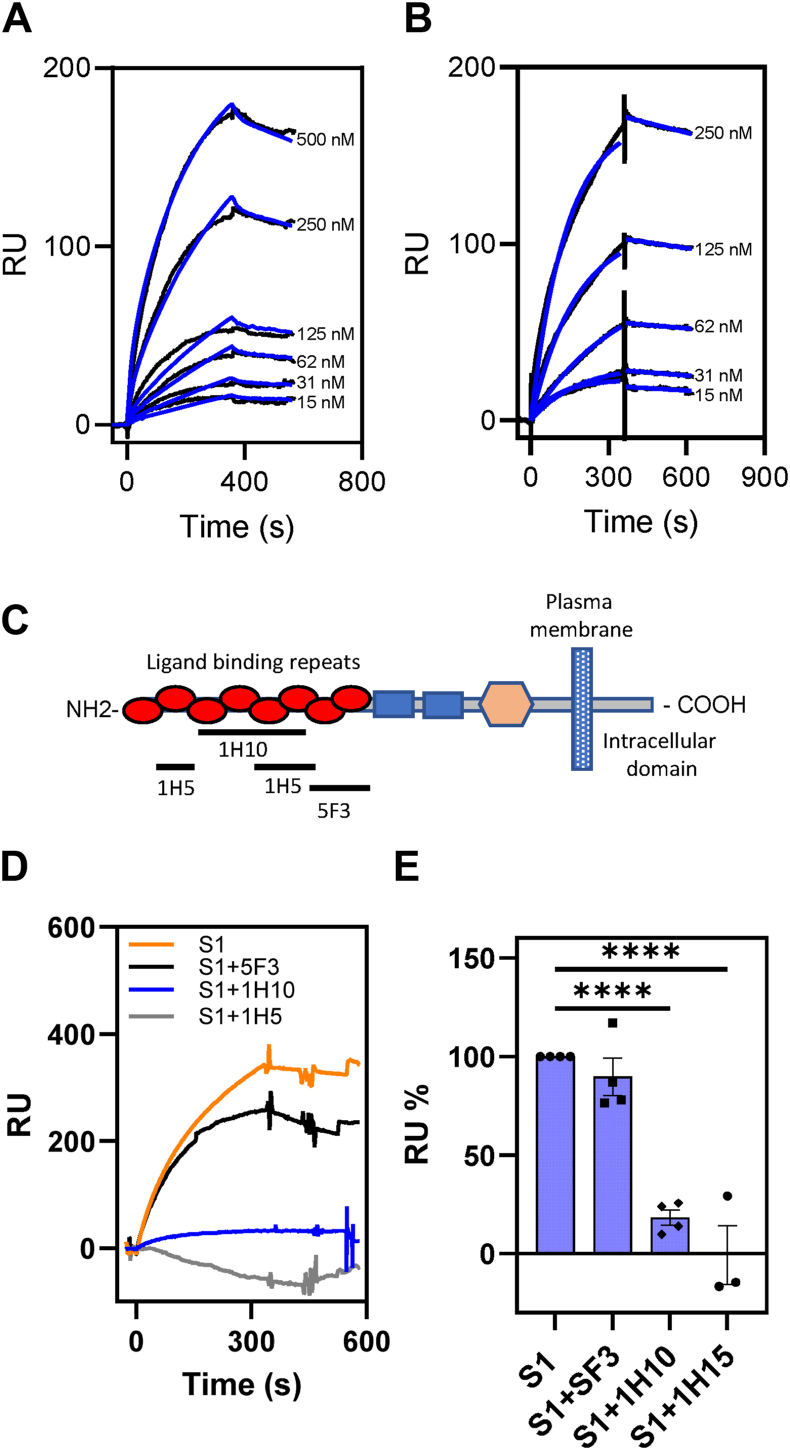
**The S1 subunit of the SARS-CoV-2 spike protein also binds LRP2/gp330 and the VLDLR.***A* and *B*, increasing indicated concentrations of S1 subunit of SARS-CoV-2 spike protein were flowed over LRP2/gp330 (*A*) or VLDLR (*B*) coupled Biacore CM5 sensor chips. Fits of the experimental data (*black lines*) to a 1:1 Langmuir binding model are shown as *blue lines*. The data are representative of three independent experiments. *C*, domain organization of the VLDLR showing location of monoclonal antibody binding. *D*, inhibition of S1 subunit of the SARS-CoV-2 spike protein (250 nM) binding to VLDLR coupled CM5 sensor chip by 200 nM of various VLDLR monoclonal antibodies. *E*, replicates of antibody inhibition experiments represented as % of S1 binding (means ± SEM, One-Way ANOVA with Dunnett’s Multiple comparisons test ∗∗∗∗*p* < 0.001).

Several members of the LDL receptor family also bind ligands that are recognized by LRP1. These include the VLDL receptor (20) and LRP2/gp330 (21). To determine if these receptors also recognize the SARS-CoV-2 spike protein, we quantified the binding of this protein to SPR chips coated with soluble forms of LRP2 (Fig. 2*A*, Table 1) or recombinant soluble VLDLR (Fig. 2*B*, Table 1). The results confirm that the SARS-CoV-2 spike protein binds avidly to both receptors. We previously prepared monoclonal antibodies to the ligand-binding region of the VLDLR (22) and have mapped out the regions on the receptor which are recognized by these antibodies. This prior study revealed that monoclonal antibody 1H5 recognizes repeats 2 and 5 to 6; monoclonal antibody 1H10 recognizes repeats 3 to 6, while antibody 5F3 recognizes repeats 7 to 8 (Fig. 2*C*) (23). Using SPR co-injection techniques, we found that monoclonal antibodies 1H5 and 1H10 both block binding of the S1 subunit of the spike protein to immobilized VLDLR, whereas monoclonal antibody 5F3 has no impact on binding (Fig. 2, *D* and *E*).

### The SARS-CoV-2 receptor binding domain interacts with LRP1 via critical lysine residues

**Figure 3:**
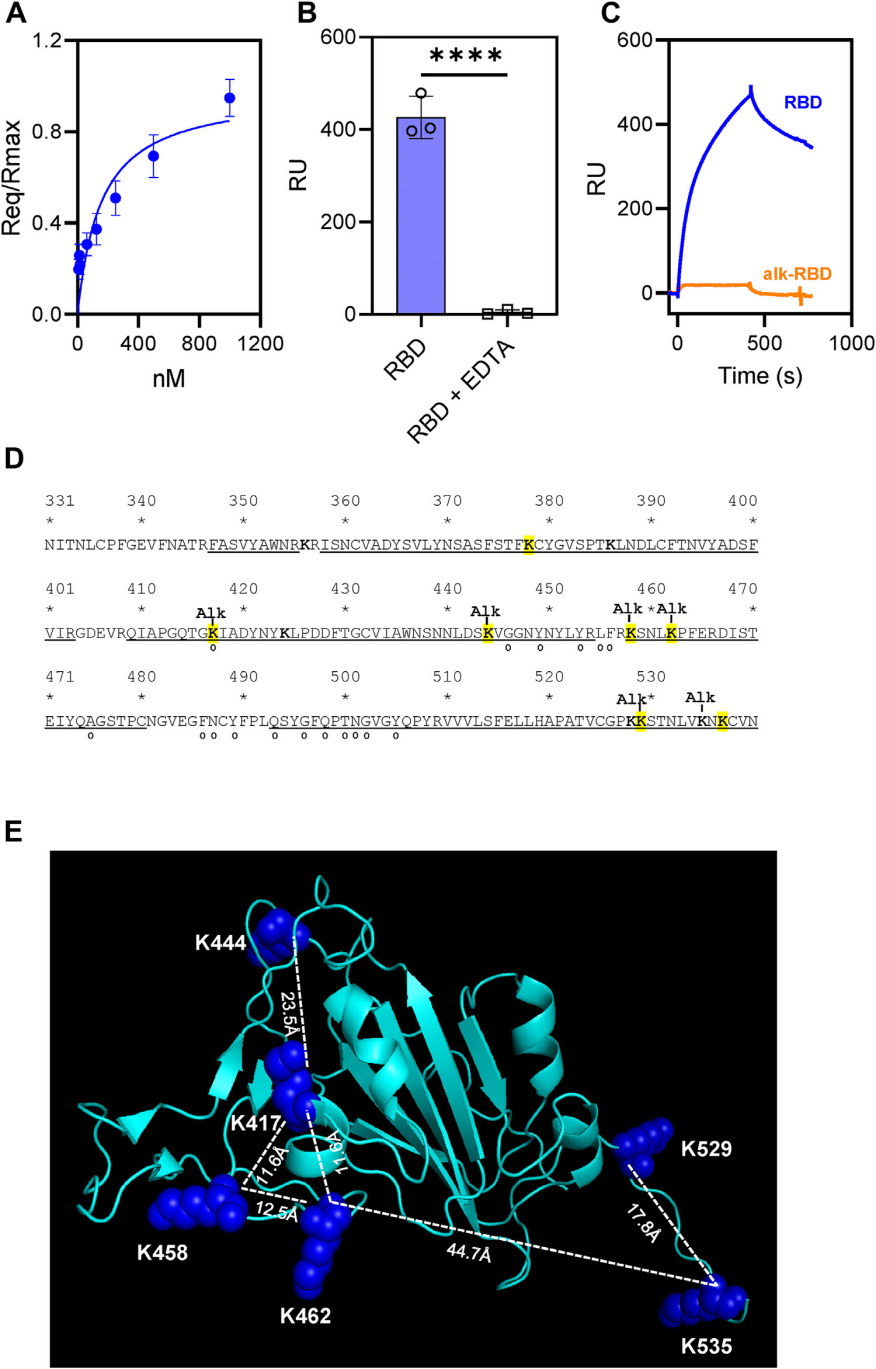
**The RBD of the S1 subunit of SARS-CoV-2 spike protein binds LRP1.***A*, equilibrium binding analysis of the binding of increasing concentrations of the RBD to an LRP1-coupled Biacore CM5 chip. Req at each concentration was obtained by fitting the data to a pseudo-first order process and the Req values is plotted vs total RBD concentration. Data are fit to a binding isotherm using non-linear regression analysis available in GraphPad Prism vs 10.3 software. (Mean ± SEM, n = 3). *B*, binding of the RBD (1000 nM) to LRP1 coupled CM5 chip in presence or absence of EDTA. (*t* test, ∗∗∗∗*p* < 0.0001). *C*, binding of RBD (1 μM) or alkylated-RBD (1 μM) to LRP1 coupled-CM5 chip. The data are representative of three independent experiments. *D*, results from mass spectral analysis of the alkylated-RBD was used to identify specific lysine residues that are alkylated. Underlined residues show the extent of coverage by mass spectral analysis. Eleven of the twelve lysine residues were detected by mass spectral analysis; only K356 was not detected. Lysines residues highlighted in *yellow* are predicted to be surface assessable based on the three-dimensional structure, while “o” identifies contact residues of the RBD that in contact with ACE2 from the published structure of the RBD and ACE2 (6moj.pdb) (63). *E*, structure of the receptor binding domains present in the trimeric spike protein in the “open” conformations. Accessible lysine residues (ASA>50%) on the receptor binding domain were alkylated and available for interaction with LRP1 are shown as blue spheres. Structure visualized using PyMOL software (7DK3.pdb).

We next investigated whether the receptor-binding domain (RBD) of the SARS-CoV-2 spike protein interacts with LRP1. Increasing amounts of RBD were flowed over an LRP1-coated SPR surface. Unlike the trimeric spike protein and S1 subunit, the kinetic data for the RBD did not fit a 1:1 Langmuir-binding isotherm. Thus, SPR equilibrium measurements were performed. The results of this experiment revealed a K_D_ value of 192 ± 59 nM (Fig. 3*A*). We note that the SARS-CoV-2 spike protein RBD did not bind to LRP1 when EDTA was included in the buffer (Fig. 3*B*), confirming the specificity of the interaction.

Several LRP1 ligands, including blood coagulation factor VIII, plasminogen activator inhibitor one and RAP interact with LRP1 *via* specific surface lysine residues that dock into acidic pockets of the LRP1 ligand binding repeats (24, 25, 26). To determine if lysine residues are involved in the interaction of the RBD and LRP1, we chemically modified lysine residues with sulfo-NHS-acetate, which forms stable, covalent amide bonds with primary amines of lysine residues. Chemical modification of lysine residues of the RBD ablates binding to LRP1 (Fig. 3*C*).

The receptor-binding domain on the trimeric SARS-CoV-2 spike protein undergoes conformational alterations that either hide (“closed” conformation) or expose amino acids (“open” conformation) involved in ACE2 binding (27). We examined published structure (27) of the RBD located on the first protomer of the trimeric spike protein exhibiting the “closed” conformation and the third protomer exhibiting the “open” conformation for surface exposed lysine residues determined by calculating the fractional surface area using GETAREA (28) and the results of this analysis are summarized in Table 2. To identify specific lysine residues that might be involved in the interaction of the spike protein RBD, we performed mass spectral analysis of the alkylated RBD. The results of this analysis are summarized in Figure 3*D*, where the 12 lysine residues present in the RBD are highlighted in bold. Mass spectral analysis identified peptides (see underlined peptides, Fig. 3*D*) that covered 11 of these residues. In this analysis, lysine 356 was not detected. However, based on the known cryoEM structures of the trimeric spike protein (27) and the isolated RBD (4), this residue is predicted to be inaccessible. Mass spectral analysis confirmed that six of the twelve lysine residues were acetylated (Table 2, Fig. S1). The degree of acetylation varied with different lysine residues (see Table 2), likely reflecting the surface accessibility of lysine residues within the RBD. Together, these results imply that K417, K444, K458, K462, K529, and K537 are potentially involved in binding to LRP1. Since K535 is not accessible in either the “closed” or the “open” conformation of the trimeric spike protein (see Table 2), and since the degree of acetylation is low for K444 (see Table 2), it is likely that these two lysine residues are not critical for LRP1 binding. In summary, the data support a role for K417, K458, K462, and K529 in LRP1 recognition. Figure 3*E* shows the structure of the spike protein RBD in the “open” conformation with the six lysine residues implicated displayed in dark blue (27). Optimal distances between lysine residues for docking into the ligand binding repeats on LDLR family members can be estimated from known structures of various ligand-binding repeats from this family of receptors, which yield a range of ∼14 to 41 Å and average of 26 Å. Most lysine residues in the RBD fall within the range of these measurements.

**Table 2:** Calculation of surface exposure of lysine residues and degree of lysine acetylation

Residue	Accessible surface area of trimeric spike protein RBD protomer “closed” conformation^a^	Accessible surface area of trimeric spike protein RBD protomer “open” conformation^a^	Accessible surface area of RBD in complex with ACE2^a^	Peptides detected by mass spectrometry^b^	Acetylated^c^	% acetylation^d^
PDB file	7df3.pdb	7dk3.pdb	6moj.pdb			
K356	42.1	47.8	39.9	Not detected	N/A	
K378	45.9	54	66.7	S371-K378 I358-K378	No	
K386	29.5	49.7	77.8	C379-K386	No	
K417	1.4	52	32.1	Q409-K417	Yes	54.8
K424	16.0	32.5	21.3	Q409-K424	No	
K444	67.5	62.6	73.4	N437-R454; V433-K444	Yes	12.1
K458	87.2	66.4	71.1	K458-R446; K458-F464	Yes	64.3
K462	51.4	91.1	87.8	S459-R466; K458-R466	Yes	66.4
K528	33.5	55.3	nd	V510-K529	No	
K529	100	100	nd	K529-K535	Yes	99.5
K535	31.3	42.6	nd	S530-N536 K529-K537	Yes	99.5
K537	41.1	70.8	nd	K529-K537	No	

a Calculated using GETAREA (28).

b Peptides detected by mass spectrometry that were used to confirm acetylation.

c Mass spectrometry analysis used to identify acetylated lysine residues.

d Degree of acetylation detected for specific lysine residue.

### LRP1-expressing cells mediate the endocytosis of SARS-CoV-2 S1 subunit

**Figure 4:**
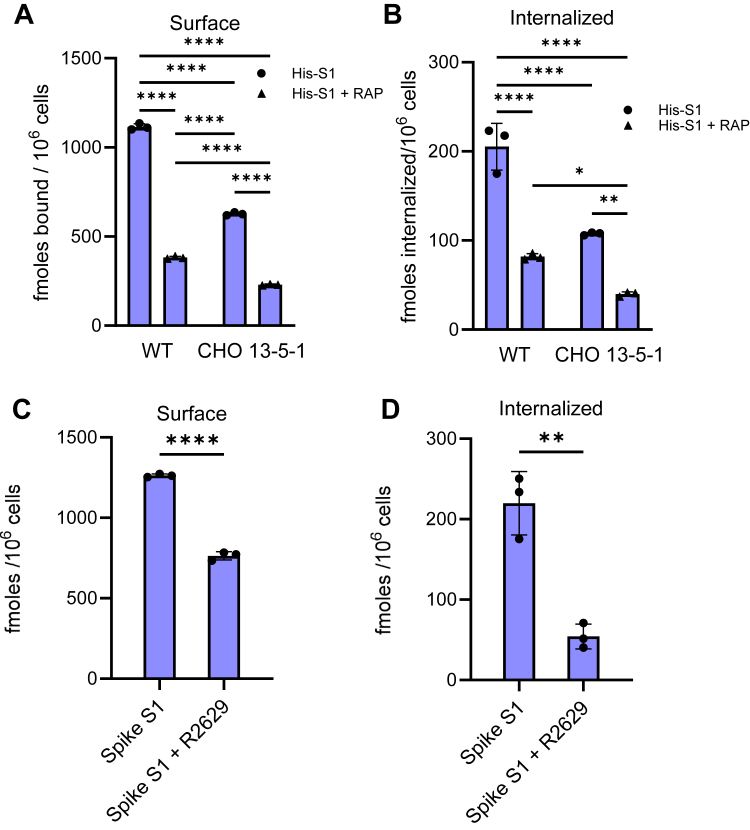
**^125^I-labled S1 subunit of the SARS-CoV-2 spike protein is internalized by LRP1.***A* and *B*, CHO WT and LRP1-deficient CHO 13-5-1 cells were incubated with 100 nM of ^125^I-labeled His-S1 subunit of the SARS-CoV-2 spike protein for 2.5 h at 37 °C in the presence or absence of 5 μM RAP. Following incubation, surface association (*A*) and internalization (*B*) were quantified. (Two-way ANOVA, Tukey’s multiple comparison test, ∗∗∗∗*p* < 0.0001; ∗∗*p* < 0.007; ∗*p* < 0.02). *C* and *D*, vascular smooth muscle cells were incubated with 100 nM ^125^I-labeled His-S1 subunit of the SARS-CoV-2 spike protein for 2.5 h at 37 °C in presence and absence of 300 μg/ml anti-LRP1 IgG (R2629). Following incubation, surface association (*C*) and internalization (*D*) were quantified. (*t* test, ∗∗∗∗*p* < 0.0001; ∗∗*p* < 0.003). The data are representative of two independent experiments, each performed in triplicate.

To confirm the SPR-binding results, cell-based experiments were performed utilizing LRP1-expressing Chinese hamster ovary (CHO) WT cells and CHO cells deficient in LRP1 (CHO 13-5-1 cells) (29). CHO WT and CHO 13-5-1 cells were incubated with ^125^I-labeled SARS-CoV-2 S1 subunit at 37 °C for 2.5 h, with or without 1 μM of RAP, an inhibitor of ligand binding by LDL-receptor family members. Following incubation, the amount of ^125^I-labeled S1 subunit bound to the cell surface (Fig. 4*A*) and internalized (Fig. 4*B*) was quantified. The results reveal that significantly more SARS-CoV-2 S1 subunits were internalized in LRP1-expressing CHO cells than LRP1-deficient CHO cells. Furthermore, significant amounts of the surface binding and internalization of the SARS-CoV-2 S1 subunit were blocked by RAP. We noticed that RAP also reduced the amount of ^125^I-labeled SARS-CoV-2 S1 subunit internalized in LRP1-deficient CHO cells. Since these cells are known to also express the VLDLR (30), we attribute the RAP-sensitive uptake to this receptor, as RAP acts as an inhibitor to this receptor as well. Overall, these results confirm that cellular forms of LRP1 can bind the SARS-CoV-2 S1 subunit and mediate its endocytosis.

We also examined the potential of LRP1 to mediate the endocytosis of ^125^I-labeled SARS-CoV-2 S1 subunit in vascular smooth muscle cells, which express large amounts of LRP1 and have been suggested as a major mediator of vascular pathology associated with SARS-CoV-2 infection (31). The contribution of LRP1 to this process was evaluated by blocking LRP1 function with anti-LRP1 IgG (R2629). The results confirm the ability of vascular smooth muscle cells to mediate internalization of ^125^I-labeled SARS-CoV-2 S1 subunit in an LRP1-dependent manner (Fig. 4, *C* and *D*).

### LRP1 expression enhances ACE2-mediated endocytosis of ^125^I-labeled SARS-CoV-2 S1 subunit

**Figure 5:**
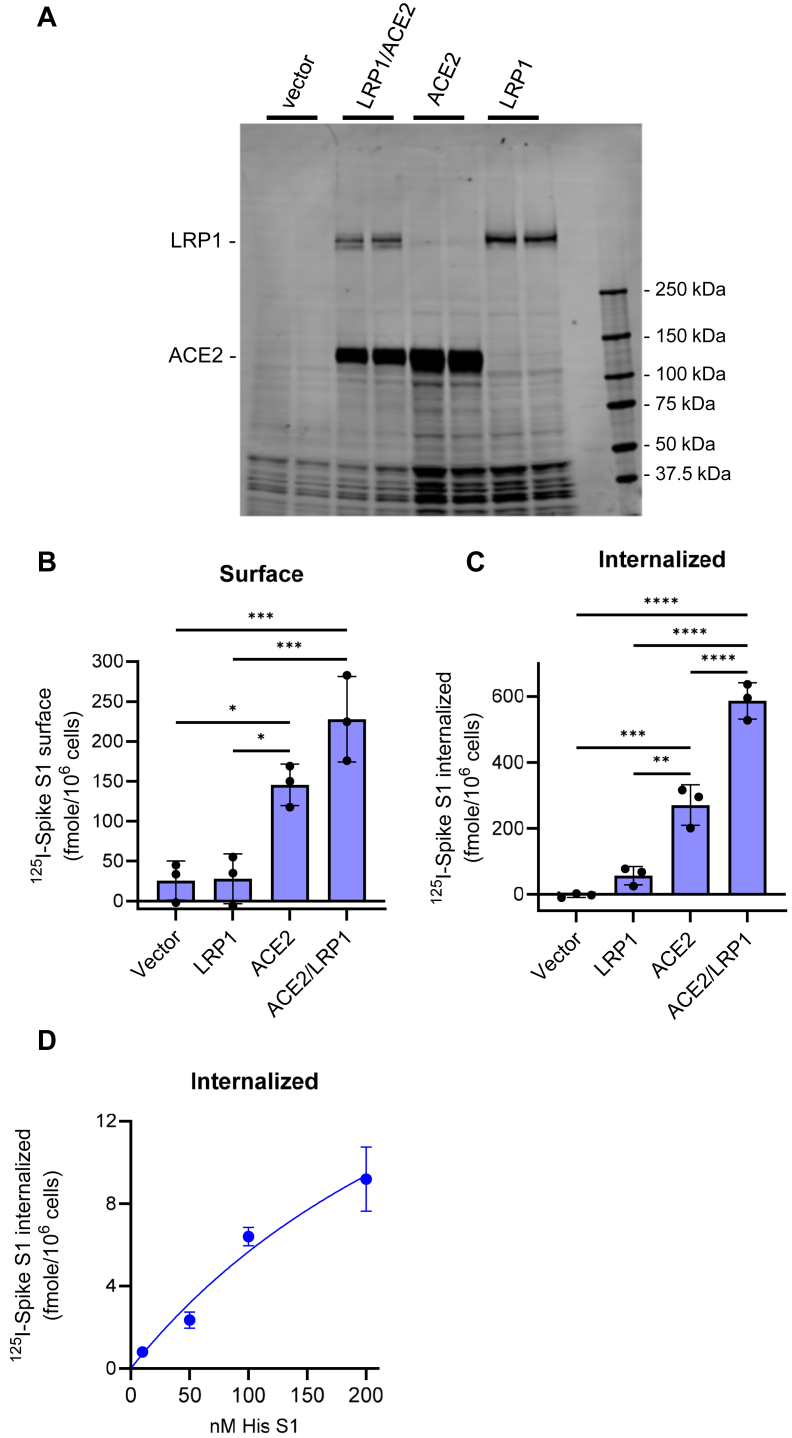
**LRP1 expression enhances ACE2 internalization of ^125^I-labeled S1 subunit of SARS-CoV-2 spike protein.** HEK293 cells were transfected with ACE2, LRP1 or ACE2 and LRP1. *A*, transfection was confirmed by immunoblot analysis using SDS-PAGE. *B* and *C*, transfected cells were incubated with 30 nM ^125^I-labeled His-S1 subunit of SARS-CoV-2 spike protein in the presence or absence of 800nM unlabeled His-S1 subunit for 2.5 h at 37 °C, and the amount of specific (excess cold subtracted) surface associated (*B*) and internalized (*C*) quantified. (One-way ANOVA with Tukey’s multiple comparison test; ∗∗∗∗*p* < 0.0001; ∗∗∗*p* < 0.006; ∗∗*p* < 0.001; ∗*p* < 0.02). *D*, HEK293T17 cells were transfected with GFP-LRP1 and the internalization of increasing concentrations of ^125^I-labeled His-S1 was quantified. LDL-receptor family member specific internalization was determined by co-incubating cells with 1 μM RAP, and the results shown represent RAP-inhibitable internalization.

The major receptor that mediates viral entry for SARS-CoV-2 is the angiotensin-converting enzyme 2 (ACE2) (2). We conducted experiments to determine if LRP1 has the potential to enhance viral entry by first examining the effect of LRP1, ACE2 and LRP1/ACE2 expression on the endocytosis of ^125^I-labeled SARS-CoV-2 S1 subunit. For these experiments, HEK293T cells were transfected with LRP1, ACE2 or co-transfected with both LRP1 and ACE2. Expression of various molecules was confirmed by immunoblot analysis (Fig. 5*A*). The ^125^I-labeled SARS-CoV-2 S1 subunit was then incubated with cells at 37 °C for 2.5 h, and the amount on the cell surface (Fig. 5*B*) and endocytosed (Fig. 5*C*) quantified. As expected, the results demonstrate a significant increase in the amount of ^125^I-labeled SARS-CoV-2 S1 subunit internalized in cells expressing ACE2. Interestingly, a significant increase in endocytosis of the ^125^I-labeled SARS-CoV-2 S1 subunit was noted in cells expressing both LRP1 and ACE2 (Fig. 5*C*), suggesting a cooperative effect between ACE2 and LRP1. We note that co-expression of LRP1 with ACE2 increases internalization of S1 subunit but does not significantly increase surface association, although there is a trend toward significance (*p* = 0.0864).

We also transfected HEK293T/17 cells with a plasmid containing LRP1 conjugated to GFP (GFP-LRP1) and examined the internalization of increasing concentrations of ^125^I-labeled S1 subunit. As controls, cells were incubated with or without 1 μM RAP to examine LDL-receptor specific internalization. The results demonstrate that S1 spike subunit protein is internalized by LDL receptors in a concentration-dependent manner (Fig. 5*D*).

### LRP1 binds soluble forms of ACE2 and colocalizes with ACE2 in transfected cells

**Figure 6:**
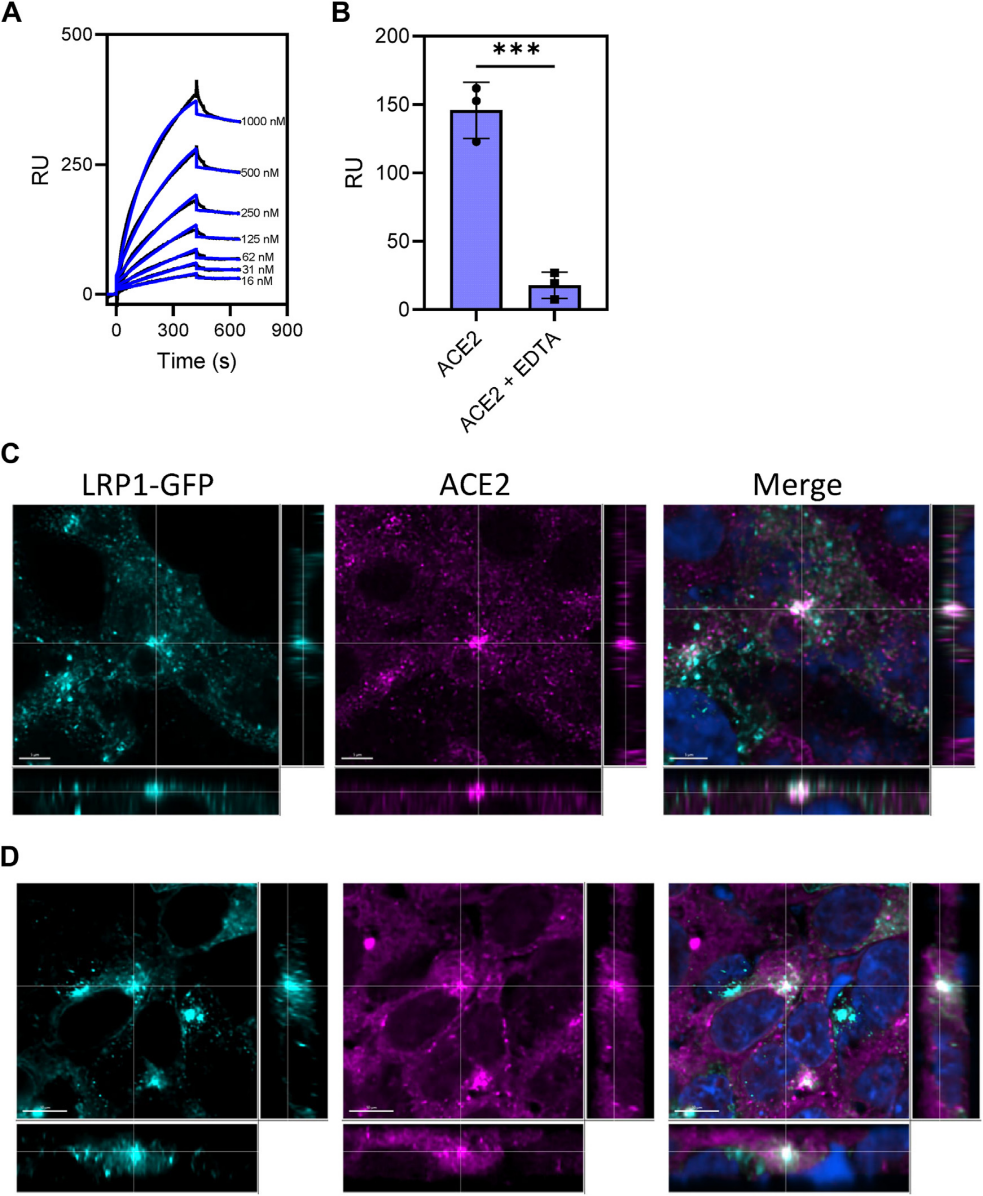
**ACE2 binds to LRP1 and colocalizes with LRP1 in transfected HEK293T cells.***A*, Increasing indicated concentrations of soluble human ACE2 were flowed over an LRP1 coupled CM5 sensor chip. Fits of the experimental data (*black lines*) to a 1:1 Langmuir binding model are shown as *blue lines*. The data are representative of three independent experiments. *B*, binding of ACE2 (125 nM) to LRP1 coupled with CM5 sensor chip in presence and absence of EDTA. (*t* test, ∗∗∗*p* < 0.006). *C* and *D*, HEK293T cells stably transfected with ACE2 were transfected with GFP-LRP1. Cells were incubated in absence (*C*) or presence of 100 nM SARS-CoV-2 spike protein (*D*). Following fixing and staining with anti-ACE2 IgG, confocal microscopy of ACE2 (*magenta*) and GFP-LRP1 (*teal*) was performed. DAPI staining is shown in *blue*. Imaris Bitplane software was used to generate the images. Scale bar in C = 5 μM; scale bar in D = 10 μM.

ACE2 is a transmembrane metalloproteinase and mediates the internalization of SARS-CoV-2 S1 subunit. Since LRP1 binds a number of metalloproteinases such as MMP1 and MMP9 (32, 33), we conducted SPR experiments to determine whether LRP1 can directly interact with the ACE2 receptor. SPR experiments revealed that soluble forms of human ACE2 directly binds to LRP1 in a concentration-dependent manner with a K_D_ of 44 ± 6 nM (Fig. 6*A*). The specificity of the interaction was confirmed by demonstrating that the presence of EDTA ablated the binding of ACE2 to LRP1 (Fig. 6*B*).

To determine if LRP1 co-localizes with ACE2 in HEK293 cells, a plasmid expressing LRP1-GFP was transfected into HEK293T cells stably transfected with ACE2. Confocal microscopy experiments revealed that LRP1 does colocalize with ACE2 in these cells before (Fig. 6*C*) or after addition of SARS-CoV-2 spike protein (Fig. 6*D*).

### LRP1 expression enhances endocytosis of SARS-CoV-2 spike protein-containing pseudovirions

**Figure 7:**
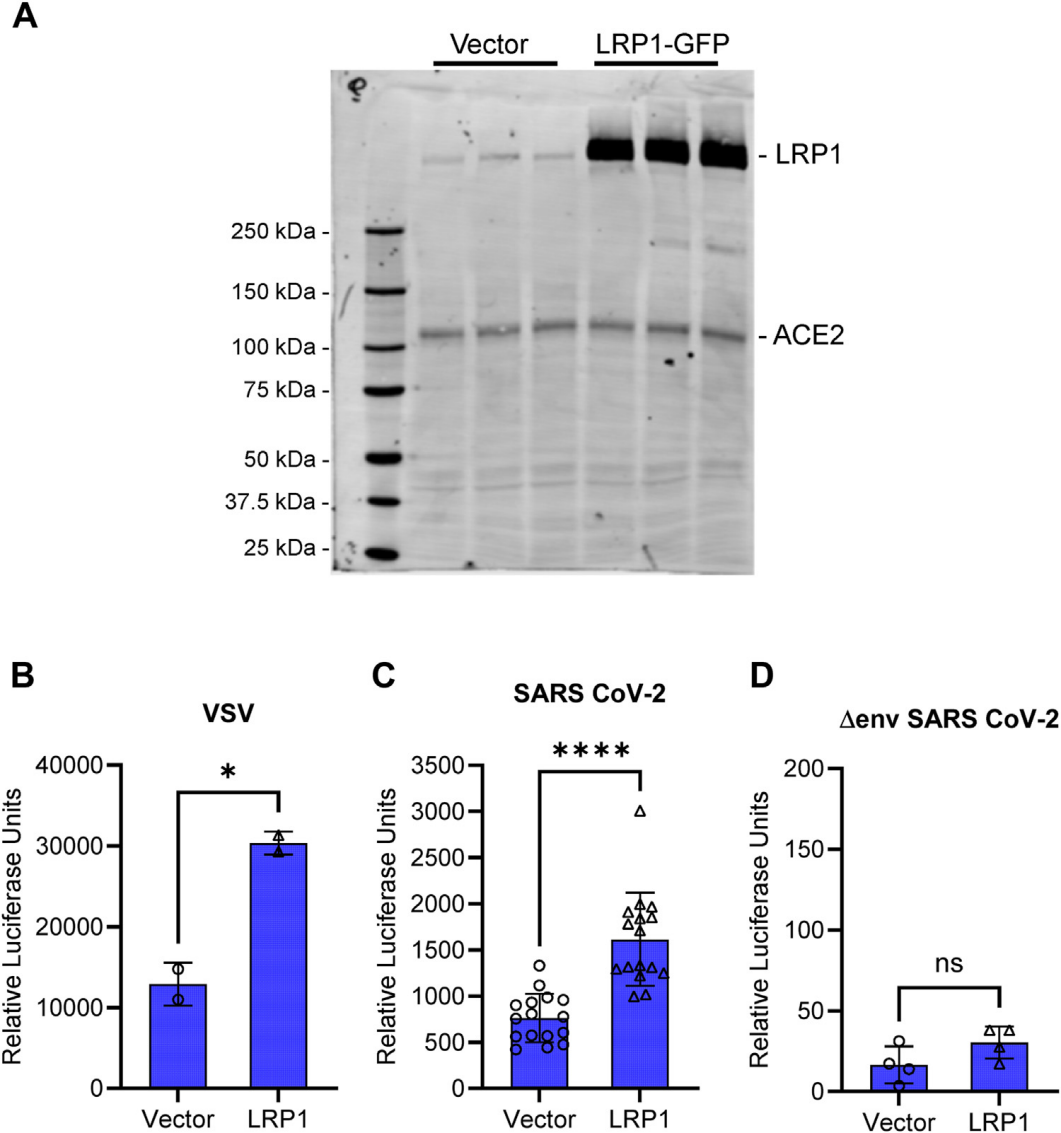
**LRP1 expression enhances ACE2-mediated internalization of SARS CoV-2 pseudovirions.** HEK293 cells stably expressing ACE2 were transfected with GFP-LRP1, and transfection was confirmed by immunoblot analysis (*A*). *B*–*D*, LRP1-transfected cells were incubated with pseudovirions containing Vesicular Stomatitis Virus (VSV) (*B*), SARS-CoV-2 S-protein (*C*) or Δenv pseudovirions (*D*). (Students *t* test; ∗*p* < 0.05, ∗∗∗∗*p* < 0.0001).

To further interrogate the involvement of LRP1 in SARS-CoV-2 spike-protein mediated viral entry, we employed a well characterized method to quantify cellular entry of viruses that utilizes pseudovirion particles containing a murine leukemia virus core and luciferase reporter that express the SARS-CoV-2 spike protein on their surface (34). These particles provide an excellent surrogate of the native virus for studying viral entry into host cells. To investigate the involvement of LRP1 in this process, HEK293T cells stably overexpressing ACE2 (HEK293T + ACE2) were either transfected with a GFP expressing construct or a GFP-tagged full-length LRP1 construct. Expression of ACE2 and LRP1 was confirmed by immunoblot analysis (Fig. 7*A*). These cells were then incubated with pseudovirions produced and collected as described (34). As a positive control for this experiment, we employed pseudovirons expressing the vesicular stomatitis virus (VSV), as LRP1 and other LDL receptor family members are known to be the host receptors for this virus (14) (Fig. 7*B*). Importantly, we noted a 2-fold increase in the amount of spike-containing pseudovirions internalized in the presence of LRP1 when compared to the GFP vector control (Fig. 7*C*). As a negative control for this experiment, we examined the endocytosis of pseudovirons lacking viral envelop proteins (Δenv) and observed little or no LRP1- or ACE2-mediated endocytosis of these particles (Fig. 7*D*). These results show that expression of LRP1 enhances the internalization of these spike-containing pseudovirions and confirms the involvement of LRP1 in facilitating pseudovirion entry.

## Discussion

In the case of SARS-CoV-2, engagement of the trimeric spike protein with cellular receptors is an important mechanism for viral entry that ultimately allows the viral genome to reach the cell cytoplasm. The cellular receptor for SARS-CoV-2 and other members of the coronavirus family has been identified as ACE2 (2, 3). However, the role of ACE2 in SARS-CoV-2 cell entry may be cell specific, as studies have found ACE2-independent internalization of SARS-CoV-2 (35). Further, employing stringent immunohistochemical analysis, Hikmet *et al.* (36) found no or low expression of ACE2 in the human respiratory system, a system dramatically impacted by SARS-CoV-2 infection. To date, a number of additional molecules have been identified that facilitate SARS-CoV-2 cell entry, which include neurophilin-1 (37), Scavenger Receptor Class B Member 1 (SCARB1 gene) (38), heparin sulfate proteoglycans (39), and CD147 (BSG/Basigin or EMMPRIN) (40). Recent studies using a haploid insertion-mutagenized mouse embryonic library raised the possibility that LRP1 may also represent an auxiliary host factor for SARS-CoV-2 infection (11).

Our objective in the current study was to investigate the hypothesis that LRP1 contributes to SARS-CoV-2 viral entry. Using surface plasmon resonance measurements, our data reveal that the SARS-CoV-2 trimeric spike protein as well as its S1 subunit binds tightly to LRP1, with K_D_ values similar to those found for the interaction of the SARS-CoV-2 S1 subunit with ACE2, which range from 1.2 to 94 nM (2, 17, 41). Further, we show that cells expressing LRP1 mediate the endocytosis of ^125^I-labeled S1 subunit of SARS- CoV-2, and that LRP1 expression enhances ACE2-mediated endocytosis of this protein. Finally, we demonstrate that LRP1 expression substantially increases the ACE2-mediated endocytosis of pseudovirion particles containing a luciferase reporter gene with the SARS-CoV-2 spike protein on their surface. Together, these results identify the potential of LRP1 to enhance ACE2-mediated SARS-CoV-2 virus entry.

**Figure 8:**
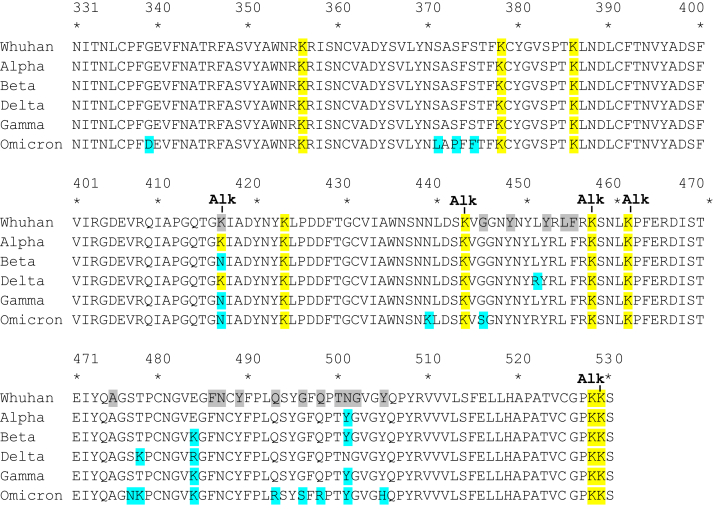
**Alignment of the RBD of the SARS-CoV-2 spike protein from various mutants.** Changes in the amino acid present in various mutants is highlighted in *blue*. Amino acid residues in contact with ACE2 are highlighted in *grey*. Conserved lysine residues are highlighted in *yellow*. Surface exposed lysine residues that were alkylated and therefore potentially available for binding LRP1 are highlighted with Alk.

LRP1 was originally identified as the hepatic receptor responsible for the removal of α_2_M-protease complexes from the liver (42, 43). This large receptor functions as a highly efficient endocytic and signal transducing receptor that plays an important role in regulating vascular development, lipoprotein metabolism, and inflammation (44, 45, 46). LRP1 is widely expressed in most tissues and cell types, and its expression is particularly abundant in hepatocytes, adipocytes, neurons, vascular smooth muscle cells, fibroblasts and macrophages. LRP1 interacts with over 70 structurally unrelated ligands, and while structural information is somewhat limited at present, data is emerging suggesting the importance of exposed surface lysine residues on ligands that, at least in part, mediate interaction of ligands with LRP1. This concept emerged from random mutagenesis studies revealing that lysine residues 256 and 270 in the RAP D3 domain is required for its binding to LRP1 (47) and from the structure of the RAP D3 domain in complex with two LDLa repeats from the LDL receptor which found these two lysine residues encircled by three conserved acidic residues located on the receptor (48). We found that chemical modification of lysine residues in the SARS-CoV-2 spike protein RBD with Sulfo-NHS-Acetate prevented the binding of this molecule to LRP1, implying that surface available lysine residues on the SARS-CoV-2 RBD are important for its interaction with LRP1. Mass spectral analysis of acetylated RBD suggests the involvement of K417, K444, K458, and K529 on the RBD as potential candidate lysine residues important for interacting with LRP1. Following the initial identification of the SARS-CoV-2 virus, it became evident that the evolution of this virus resulted in the emergence of mutations that impacted the virus transmissibility as well and antigenicity. Several of these lysine residues are conserved (Fig. 8). Several of these mutations occur within the spike protein and not only impact antigenicity of the virus but receptor recognition as well (49). For example, recent studies employing surface plasmon resonance have confirmed increased affinity of the RBD from the Alpha, Beta, Gamma, and Delta SARS-CoV-2 variants with human ACE2 (50). Currently, it is not known how these mutations in variants impact LRP1 binding.

Our studies also indicate that LRP1 directly binds soluble forms of ACE2 *in vitro*. Immunofluorescence studies confirmed some colocalization of LRP1 and ACE2 in transfected HEK293T cells. ACE2 is a type I transmembrane zinc metalloproteinase that regulates the renin–angiotensin system, a system that modulates cardiovascular and renal physiology. In this capacity, ACE2 functions by converting the vasoconstrictive peptide Angiotensin II (Angiotensin 1–8) to Angiotensin 1 to 7 and thus attenuates Angiotensin II signaling pathways. Excessive Angiotensin II mediated-signaling is known to contribute to the development and progression of aortic aneurysms and dissections (51, 52, 53). A decrease in ACE2 function shifts the balance to the Angiotensin II-mediated signaling pathway resulting in disease progression, while increased ACE2 function shifts the balance to the Angiotensin 1 to 7 signaling axis leading to protection from disease (51, 52, 53). Interestingly, we observed spontaneous formation of aneurysms in the superior mesenteric artery in mice in which LRP1 was genetically deleted in vascular smooth muscle cells (smLRP1−/− mice) (54). Proteomic analysis of the vessels from smLRP1−/− mice revealed activation of Angiotensin II-mediated signaling pathways, and we observed that treatment of smLRP1−/− mice with losartan, an angiotensin II type I receptor (AT_1_R) antagonist, or use of angiotensinogen antisense oligonucleotides (ASO) to reduce plasma angiotensinogen levels restored the SMA phenotype in smLRP1−/− mice and prevented aneurysm formation (54). These results reveal that LRP1 attenuates Angiotensin II-mediated signaling pathways. While at present, the molecular mechanism by which LRP1 regulates Angiotensin II-mediated signaling is not understood, it is intriguing to consider that this may occur *via* coordination of LRP1 with ACE2 function.

Our surface plasmon resonance studies also noted that the SARS-CoV-2 spike protein binds avidly to two other members of the LDL receptor family, the VLDLR and LRP2/gp330. The VLDLr is expressed in the brain, skeletal muscle and the heart, with lower amounts in adipose tissue, kidney, ovary, testis and lung (55), while LRP2/gp330 is primarily expressed in epithelial cells that include the kidney proximal tubules, type II pneumocytes in the lung, the parathyroid, thyroid, epididymis, uterus, retina and ciliary epithelium of the eye (56). While we did not explore the significance of the interaction of spike protein of SARS-CoV-2 with the VLDLR and LRP2 beyond surface plasmon resonance measurements in the current study, it is possible that these receptors also are able to participate in internalization of this virus as well. Since multiple organs have been shown to be vulnerable to SARS-CoV-2 infection outside of the respiratory system, it is important to recognize that other mechanisms by which this virus can enter host cells in these organs exists. Specifically, in the context of LRP2, both ACE2 (57) and LRP2 are highly expressed in kidney proximal tubule cells and may also cooperate to mediate the endocytosis of SARS-CoV-2 into host cells. Furthermore, with the prevalence of long COVID and persistent cognitive impairment associated with COVID-19, it is important to note that both LRP1 and LRP2 have been shown to endocytose ligands across the blood–brain barrier. For example, LRP2, is apically expressed and capable of transporting ligands across the blood-brain barrier (58). Further, LRP1 is a key receptor for in Aβ transport and mediator of its transcytosis across the BBB (59). The diverse biological functions of various LDL receptor family members implicate further possible involvement of these receptors in the endocytosis of virus into host cells of various organs.

In summary, we have obtained evidence identifying LRP1 as a receptor that impacts SARS-CoV-2 cellular entry. While most studies have exclusively focused on the SARS-CoV-2/ACE2 interaction, it has become clear that other mechanisms exist by which SARS-CoV-2 enters cells. Our studies imply that LRP1 cooperates with ACE2 in mediating endocytosis of pseudovirion particles expressing the SARS-CoV-2 spike protein on their surface. The potential involvement of members of the LDL receptor family in SARS-CoV-2 entry into cells would give insight into the widespread physiological impacts this virus has on human populations.

## Experimental procedures

### Proteins

The SARS-CoV-2 trimeric spike protein, obtained from the Bioexpression and Fermentation Facility of the University of Georgia, was stabilized in prefusion conformation by incorporating proline substitutions at residues 986 and 987 in the C-terminal S2 fusion machinery and by converting the furin cleavage site (residues 682–685) to Gly-Ser-Ala-Ser (17). The SARS-CoV-2 spike protein S1 subunit (Val16-Arg685) expressed as a fusion with the Fc region of mouse IgG1 at the C-terminus was purchased from Sino Biological (40,591-V05H1). The SARS-CoV-2 spike protein S1 subunit (Val16-Arg685) expressed with a His tag at the C-terminus was purchased from Exonbio (19Cov-S110). The SARS-CoV-2 spike protein receptor binding domain (his tagged, containing residues Arg319-Phe541) was purchased from R&D systems (Catalog number 10523-CV-100). Recombinant human angiotensin-converting enzyme two ectodomain (Gln18 – Ser740) was purchased from RayBiotech (230–30165). LRP1 was purified from human placenta as described (42). Soluble forms of the VLDL receptor containing repeats 1 to 8 was prepared as described (22). LRP2/gp330 was purified from rat kidneys as described (60). RAP was prepared as described (19). LRP1 clusters II, III, and IV were purchased from Innovative Research. VLDLR antibodies were prepared in house as described (22). Rabbit antibodies (R2629) prepared in house against purified LRP1 have been described (61).

### Alkylation of the spike protein RBD and mass spectral analysis

The RBD was incubated with 50-fold molar excess (over lysine residues in RBD) of Sulfosuccinimidyl Acetate in PBS. The reaction was allowed to continue for 3 h at 4 °C. The alkylated RBD was then dialyzed against PBS overnight at 4 °C. Mass spectral analysis was carried out by PTM BIO LLC . An aliquot of the alkylated RBD was reduced, alkylated, and digested with trypsin overnight at 37 °C. Post-digestion, samples were eluted and dried *via* SpeedVac, and dried samples were reconstituted in Mobile Phase A (97.9% H_2_O, 2% Acetonitrile, 0.1% formic acid). Samples were then injected into a homemade reverse phase C18 analytical column on Thermo Vanquish Neo UHPLC system. The gradient was comprised of 4% mobile phase B (80% Acetonitrile, 0.1% Formic Acid) for 0.5 min at a constant flow rate of 0.9 μl/min, 4 to 8% for 0.1 min at a decreased flow rate to 0.4 μl/min, 8 to 25.5% for 50 min, 25.5 to 35% for 6.9 min, 35 to 55% for 0.5 min at an increased flow rate to 0.8 μl/min, 55 to 99% for 0.5 min at an increased flow rate to 0.9 μl/min, then held at 99% for 1.5 min. The peptides were subjected to NSI source followed by tandem mass spectrometry (MS/MS) in Thermo Orbitrap Astral. The electrospray voltage applied was 2.0 kV. The Full Scan m/z scan range was set to 380 to 980, Automatic Gain Control (AGC) of 500%, maximum IT of 50 ms, on the Orbitrap detector at a resolution of 240,000. DDA MS/MS m/z scan range was set to 150 to 2,000, isolation window of 1.6 m/z, CE of 29%, AGC of 500%, maximum IT of 20 ms, on the Astral detector. Post acquisition, data were analyzed with BioPharma Finder *v*5.2. The maximum number of modifications was set to 3: carbamidomethylation, oxidation (MW), and acetylation (K) were set as variable modifications. For peptide identification, the maximum peptide mass was set to 11,000 Da and the mass accuracy was set to 9 ppm.

### Surface plasmon resonance

Binding of SARS-CoV-2 spike S1-mFc recombinant protein to LRP1, VLDLR, or LRP2 was assessed using a Biacore 3000 optical biosensor system (GE Healthcare Life Sciences). Full-length LRP1, purified from placenta, VLDLR 1 to 8, or LRP2 purified from rat kidneys was coupled to a Sensor Chip CM5 (GE Healthcare Life Sciences BR-1003–99) using an Amine Coupling Kit (GE Healthcare Life Sciences BR-1000–50). A separate flow cell of the CM5 sensor chip was activated and blocked with 1 M ethanolamine and served as a control surface. Various concentrations of ligand in HBS-P Buffer (0.01 M HEPES pH 7.4, 0.15 M NaCl, 0.005% (v/v) Surfactant P20; GE Healthcare Life Sciences BR100368) supplemented with 1 mM CaCl_2_ was flowed over the surface of the receptor-coupled sensor chip at a rate of 10 μl/min at 25 °C. To account for flow cell-dependent refractive index changes, sensorgrams of HBS-P Buffer only injections were subtracted from the sensorgrams for each of the respective ligand concentrations. To confirm ligand binding to LRP1, 1 μM receptor associated protein (RAP), an LRP1 antagonist, was co-injected with 500 nM S1 subunit and flowed over the surface of the LRP1-coupled sensor chip. Similarly, 200 nM 1H5, 1H10 or 5F3, monoclonal antibodies against VLDLR, was co-injected with 250 nM S1 subunit and flowed over the surface of the VLDLR-coupled sensor chip. Calcium-dependent ligand binding to LRP1 was assessed by flowing various concentrations of ligand in HBS-EP buffer (0.01 M HEPES pH 7.4, 0.15 M NaCl, 3 mM EDTA, 0.005% v/v Surfactant P20; GE Healthcare Life Sciences BR100188) over the LRP1-coupled sensor chip. Between sample runs, sensor chip surfaces were regenerated with 15 s injections of 100 mM phosphoric acid at a flow rate of 100 μl/min. The kinetic data were fit to a 1:1 Langmuir binding equation using BiaEvaluation 3.02 software. For equilibrium binding analysis, the association data were fit to a pseudo-first order process to determine maximum response units at equilibrium (R_eq_). The equilibrium dissociation constant (K_D_) was then determined by plotting R_eq_ values *versus* ligand concentration and fitting the data to a single class of sites using non-linear regression analysis (GraphPad Prism vs10; San Diego, CA). All SPR experiments were repeated at least three times.

### Cells

Wild-type Chinese hamster ovary (CHO WT) cells and LRP1-deficient Chinese hamster ovary (CHO 13-5-1) cells (29) were cultured in Dulbecco’s Modified Eagle’s Medium/Hams F-12 50/50 Mix (DMEM/F-12; Corning 10–090-CV) supplemented with 10% fetal bovine serum (FBS; Atlanta Biologicals S12450H) and penicillin-streptomycin (P/S; Corning 30–002-CI) and maintained at 37 °C, 5% CO_2_ in a humidified atmosphere. HEK293T/17 cells were purchased from ATCC. Cells were cultured in Dulbecco’s Modified Eagle’s Medium supplemented with 10% fetal bovine serum and penicillin-streptomycin and maintained at 37 °C, 5% CO_2_ in a humidified atmosphere. HEK293T stably transfected with ACE2 were provided by BEI Resources/ATCC Biorepository (NR-52511). Cells were cultured in Dulbecco’s Modified Eagle’s Medium (DMEM 1x) supplemented with 10% fetal bovine serum and penicillin-streptomycin and maintained at 37 °C, 5% CO_2_ in a humidified atmosphere. Human aortic smooth muscle cells were purchased from Lonza Bioscience and cultured in Human Vascular Smooth Muscle Cell Basal Medium (Gibco, REF M–251–500).

### Immunofluorescence

HEK293T cells stably transfected with ACE2 cells were grown on 18-chamber microscope slides (Ibidi μ-slide 18 Well ibiTreat, REF 81816) in DMEM supplemented with 10% FBS, seeded at 2000 cells/well. Cells were either transfected using Lipofectamine 3000 (Thermo Fisher Scientific) with either an empty GFP-only construct or a GFP-tagged full-length LRP1 construct for 48 h. Cells were then incubated in PBS or 100 nM Spike S1 subunit protein (Exon Bio, Catalog No.19Cov-S110) for 24 h. Cells were washed with DPBS and then fixed in 4% paraformaldehyde for 30 min at room temperature. Cells were permeabilized in 0.2% Triton-X 100 in PBS for 15 min at room temperature. Slides were washed with DPBS and blocked in donkey serum in wash buffer (1% BSA + 0.2% Tween 20 in PBS). After blocking, cells were treated with anti-ACE2 antibody (Invitrogen ACE2 Recombinant Rab maB SN0754, REF MA5-32307) overnight at 4 °C. Cells were then incubated in Goat anti-rabbit IgG AlexaFluor Plus 647 (Invitrogen, REF A32733). VECTASHIELD HardSet Antifade mounting media (DAPI, Vector Laboratories, H-1500) was used to stain the nuclei. Fluorescent images were acquired using a CSU-W1 spinning disk confocal system (Nikon) in the Center for Innovative Biomedical Resources Confocal Microscopy Facility at the University of Maryland School of Medicine. Images were acquired with a 40x oil-immersion objective as z-stacks with a step size of 0.5 μm. Imaris Bitplane 10.2 was used to generate images for publication and analyze regions of colocalization.

### Cell-mediated internalization assays

SARS-CoV-2 spike His-S1 subunit was labeled with the iodine-125 isotope (^125^I; PerkinElmer NEZ-033) using Pierce Iodination Reagent (Thermo Scientific 28,600). Iodinated S1 (^125^I-S1) was desalted using a PD-10 Sephadex G-25 column (GE Healthcare 17–0851–01). CHO WT and CHO 13-5-1 cells used in cell-mediated internalization assays were trypsinized, resuspended in DMEM/F-12, 10% FBS, P/S and seeded in 12-well tissue culture plates. Cultures were maintained overnight for 16 to 18 h. Cultures at 60% confluency were washed with DPBS and incubated in serum-free DMEM, 20 mM HEPES, 1 mM CaCl_2_, 1.5% bovine serum albumin (BSA; Sigma-Aldrich A7030) (assay media) for 1 hour at 37 °C. After 1 hour, assay media was aspirated from each well and cells were incubated with 500 μl of assay media containing 100 nM ^125^I-labeled S1 subunit, with or without 5 μM RAP. The incubation was carried out for 2.5 h at 37 °C. Following incubation the assay media was removed, cells were washed twice with DPBS, and the wash was carefully aspirated. Cells were then detached from the plate with 0.05% trypsin, 0.53 mM EDTA (Corning 25–052-CI) containing 50 μg/ml proteinase K (Thermo Scientific 17,916), collected from each well and transferred to a microcentrifuge tube, and centrifuged at 1200 rpm for 5 min at room temperature. The supernatant was then removed, and the cells were washed with 0.1 M glycine, pH 2.5. Experiments in which ^125^I-S1 subunit of SARS-CoV-2 were incubated with cells at 4 °C to prevent endocytosis confirmed that the sequential trypsin/proteinase K treatment followed by low pH wash removed 100% of ^125^I-S1 subunit from cells. The amount of ^125^I-S1 SARS-CoV-2 subunit that remained in the cell pellet was considered to be internalized. The total number of cells was counted in three separate wells and the average cell number was used for normalization. Similar procedures were followed for internalization assays using the VSMCs as well as co-transfected HEK293T cells with empty vector, LRP1 and ACE2 for internalization assay experiments.

### Pseudovirion (PV) production

Pseudovirions were prepared by adaptation of the procedure developed by Millet *et al.* (34). HEK293T/17 cells were seeded to 80 to 90% confluency in complete Dulbecco’s Modified Eagle’s Medium in 6-well tissue culture plates containing 2 ml of complete DMEM-C. Plates were incubated overnight at 37 °C. Following plating, cells are co-transfected with the following plasmids: pCMV-MLVgagpol MLV gag and pol encoding plasmid (300 ng), pTG-Luc transfer vector with luciferase reporter (400 ng), pcDNA-SARS-CoV-2 or control plasmids (empty vector or VSV) (300 ng). Plasmids were generously provided by Gary Whittaker (Cornell University). PEI was used as a transfection agent with a ratio of transfection reagent: plasmid DNA of 3:1. Reagent and medium were mixed and sat at RT for 20 min in a 1:1 ratio mix. Media was aspirated off cells, and 100 μl of transfection mix was dropped in a circular motion with 1 ml of pre-warmed reduced serum cell culture medium per well. Another 1 ml of medium without antibiotics is added to the well and incubated overnight. The next day, the media was transferred to a 50 ml conical centrifuge tube and centrifuged at 290*g* for 7 min to remove cell debris. Aliquots (1 ml) of pseudotyped particles present in the media were stored for future use at −80 °C.

### Infectivity assays

Infectivity assays were performed as described (34). HEK293T + ACE2 cells were plated on gelatin-coated 24-well plates at a seeding density of 125,000 cells/well with DMEM media with 10% FBS with no antibiotics present. Cells were grown overnight to roughly 60% confluency and transfected with LRP1-expressing plasmids or empty vector as controls. The plates were then incubated overnight at 37 °C and washed three times with 0.5ml pre-warmed DPBS. After the final wash, 200 μl of thawed pseudotyped particles were added to the cells, and the volume was adjusted to 500 μl with media. Incubation was carried out for 24 h at 37 °C prior to conducting the luciferase assay, which used Promega Luciferase Assay Systems (Catalog No. PR-E4550) according to the manufacturer’s protocol. Briefly, 100 μl of luciferase assay lysis buffer was added to cells infected with pseudotyped particles. Cells were then incubated for 15 min while rocking at room temperature. Twenty microliter of luciferin substrate was then added, and luciferase activity measurement was performed on FlexStation three by endpoint kinetic measurement.

### SDS-PAGE and immunoblot analysis

Cell cultures were collected in modified RIPA lysis buffer and analyzed by immunoblotting as previously described (62). Equal amounts of protein from each sample were mixed with loading buffer and resolved by electrophoresis on a Novex four to 12% Tris-Glycine Mini Protein Gel. This was then transferred to polyvinylidene difluoride membranes for Western blot analysis. Membranes were blocked with Odyssey blocking buffer and incubated with rabbit antiLRP1 R2629 IgG at 1:1000, goat anti-ACE2 antibody (R&D systems Catalog #AF933) at one ug/ml, and anti-GAPDH 1:1000 overnight at 4 °C. Antibody binding to membrane was detected with IRDye 680RD anti-goat (ACE2) and IRDye 800 anti-rabbit (LRP1) (LI-COR Biosciences) at a concentration of 1:10,000. The membrane was then washed three times with 0.05% Tween-20 in Tris-buffered saline and imaged using a LI-COR Odyssey Infrared Imaging System.

### Statistics

All results are presented as mean ± SEM. Data were analyzed for significance using a Student’s *t* test or one-way ANOVA, followed by a Tukey *post hoc* test (GraphPad Prism 10). A *p*-value ≤ 0.05 was set as the threshold for significance.

## Data availability

All data is contained within the manuscript.

## Supporting information

This article contains supporting information.

## Conflict of interest

The authors declare that they have no conflicts of interest with the contents of this article.
