# Supplementary material for: The LDL receptor-related protein 1 (LRP1) facilitates ACE2-mediated endocytosis of SARS-CoV2 spike protein-containing pseudovirions
Source: J Biol Chem. 2025 May 9;301(6):110227. doi: 10.1016/j.jbc.2025.110227 (PMC12192683; doi:10.1016/j.jbc.2025.110227)
Supplement: Supplemental Figure 1 [file mmc1.pdf]

Supplemental Figure 1. Fragmentation spectra for peptides containing acetylated lysine residues. The receptor binding domain (RBD) from the SARS-CoV-2 spike protein (Arg319-Phe541) was incubated with a 50-fold molar excess over lysines of Sulfosuccinimidyl Acetate in PBS for 3 hours at 4 ºC. Following digestion with trypsin, peptides were separated on a reverse phase C18 analytical column followed by tandem mass spectrometry (MS/MS) in Thermo Orbitrap Astral. For peptide identification, the maximum peptide mass was set to 11,000 Da and the mass accuracy was set to 9 ppm. The MS/MS fragmentation spectra are shown for peptides containing lysine residues that were alklylated in the RBD (K417, K444, K458, K462, K529, K535).

K417 fragmentation

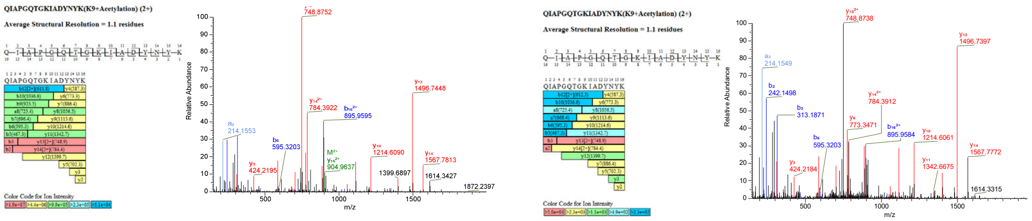

K444 fragmentation

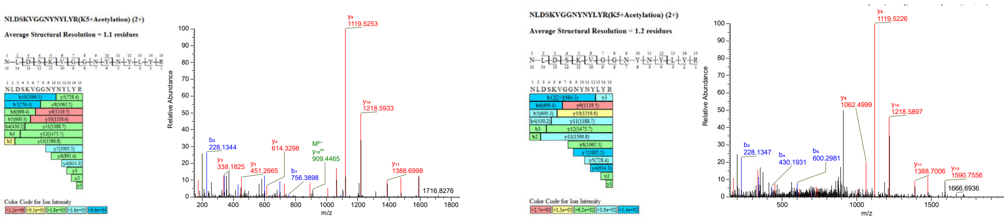

K458 fragmentation

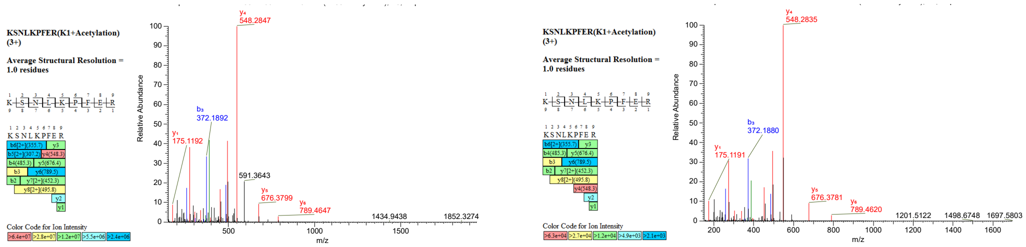

K462 fragmentation

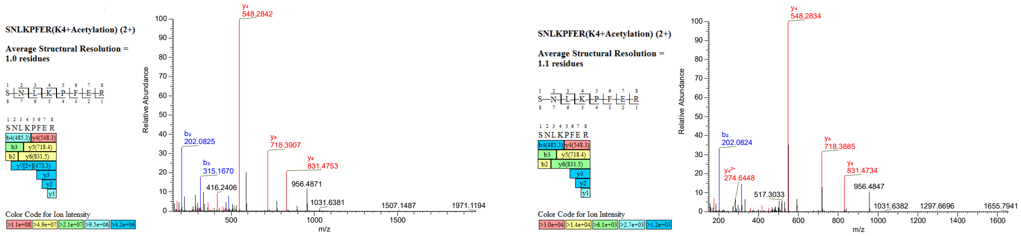

K529 fragmentation

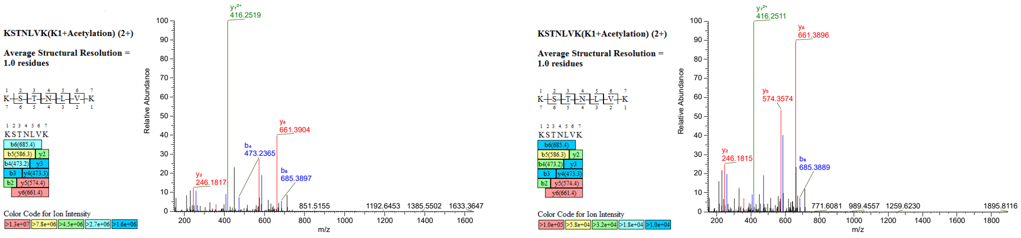

K535 fragmentation

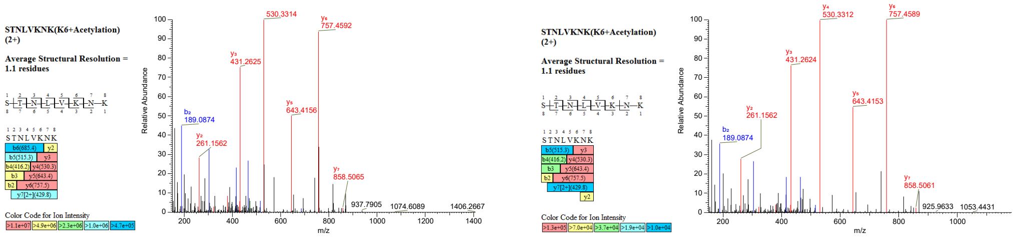

Supplemental Figure 1
